# Evaluating drivers of recent large whale strandings on the East Coast of the United States

**DOI:** 10.1111/cobi.14302

**Published:** 2024-05-29

**Authors:** L. H. Thorne, D. N. Wiley

**Affiliations:** ^1^ School of Marine and Atmospheric Sciences Stony Brook University Stony Brook New York USA; ^2^ National Oceanic and Atmospheric Administration, National Ocean Service, Stellwagen Bank National Marine Sanctuary Scituate Massachusetts USA

**Keywords:** anthropogenic threats, baleen whale, management, mortality, offshore wind, vessel strikes, vessel traffic, amenazas antropogénicas, ballena barbada, colisiones con navíos, energía eólica marina, manejo, mortalidad, tráfico de navíos

## Abstract

Anthropogenic stressors threaten large whales globally. Effective management requires an understanding of where, when, and why threats are occurring. Strandings data provide key information on geographic hotspots of risk and the relative importance of various threats. There is currently considerable public interest in the increased frequency of large whale strandings occurring along the US East Coast of the United States since 2016. Interest is accentuated due to a purported link with offshore wind energy development. We reviewed spatiotemporal patterns of strandings, mortalities, and serious injuries of humpback whales (*Megaptera novaeangliae*), the species most frequently involved, for which the US government has declared an “unusual mortality event” (UME). Our analysis highlights the role of vessel strikes, exacerbated by recent changes in humpback whale distribution and vessel traffic.  Humpback whales have expanded into new foraging grounds in recent years. Mortalities due to vessel strikes have increased significantly in these newly occupied regions, which show high vessel traffic that also increased markedly during the UME. Surface feeding and feeding in shallow waters may have been contributing factors. We found no evidence that offshore wind development contributed to strandings or mortalities. This work highlights the need to consider behavioral, ecological, and anthropogenic factors to determine the drivers of mortality and serious injury in large whales and to provide informed guidance to decision‐makers.

## Abstract (es)

Análisis de las causantes de los recientes varamientos de ballenas en la costa este de los Estados Unidos

**Resumen:**

El estrés antropogénico amenaza a las ballenas en todo el mundo. El manejo efectivo requiere comprender en dónde, cuándo y por qué ocurren las amenazas. Los datos de varamientos proporcionan información clave sobre los puntos críticos geográficos de riesgo y la importancia relativa de varias amenazas. Actualmente existe un interés público considerable por el incremento en la frecuencia de varamientos de ballenas que ocurren en la costa este de los Estados Unidos desde 2016, al cual el gobierno nacional ha denominado un “evento inusual de mortalidad” (EIM). El interés se acentúa debido a la supuesta conexión con el desarrollo de la energía eólica marina. Revisamos los patrones espaciotemporales de los varamientos, mortandad y lesiones graves de las ballenas jorobadas (*Megaptera novaeangliae*), la especie involucrada con mayor frecuencia. Nuestro análisis resalta el papel de las colisiones con navíos, agudizados por los cambios recientes en la distribución de la especie, y el tráfico de navíos. Las ballenas jorobadas se han expandido hacia nuevas áreas de forrajeo y los años recientes. La mortandad causada por las colisiones con navíos ha incrementado significativamente en estas regiones ocupadas recientemente, las cuales también muestran un tráfico elevado de navíos que también incrementó durante el EIM. La alimentación superficial y en áreas someras podrían ser factores contribuyentes. No encontramos evidencia de que la energía eólica marina contribuya a los varamientos o a la mortandad. Este trabajo resalta la necesidad de considerar los factores ecológicos, antropogénicos y de comportamiento para determinar las causas de la mortalidad y las lesiones graves en las ballenas y de proporcionar orientación informada para quienes toman las decisiones.

## INTRODUCTION

Populations of many large whales were devastated by commercial whaling, and today large whales are still affected by multiple anthropogenic stressors that can influence their population recovery (Leaper & Miller, 2011; Moore et al., 2021; Thomas et al., 2016). Particularly in coastal waters, human activities create multiple threats for large whales and other marine mammals worldwide, such as entanglement in fishing gear, pollution, disturbance (including noise), and vessel strike (Avila et al., 2018). Effective management of large whales requires an understanding of how, when, and where threats are occurring and the drivers of these threats (Torres et al., 2013; van Der Hoop et al., 2013; Wiley et al., 2011b). Assessments of strandings and of mortalities and serious injuries (MSI) of large whales provide a key means of understanding the relative importance of different threats facing large whales and geographic hotspots of anthropogenic threats (Dudhat et al., 2022; Grossi et al., 2021; Obusan et al., 2016; Peltier et al., 2019).

Recent strandings and associated mortality of large whales along the US East Coast have garnered considerable public interest, often spurred by statements in the media that wind turbines are causing the observed increase in strandings (Christenson, 2023; de la Garza, 2023; Parry, 2023; Robinson, 2023; Yeatman, 2023). The conversation to date has been dominated by unsubstantiated assertions, lacking a focus on data available to assess patterns of strandings, and has led to threats against biologists and managers responding to stranded whales (de la Garza, 2023). It has also been used to argue against the expansion of wind energy as an alternative to the burning of fossil fuels. Large whales strand regularly along the US East Coast, due to both natural and anthropogenic factors (Hayes et al., 2022; Wiley et al., 1995). In the United States, the collection of basic information on marine mammal strandings, such as the location, date, and species of individuals, has been coordinated and standardized since Title IV of the US Marine Mammal Protection Act (MMPA) established the Marine Mammal Health and Stranding Response Program. Further, levels of human‐caused MSI to marine mammals must be assessed following a reauthorization of the MMPA in 1994, providing detailed information on the anthropogenic threats to these species. The availability of strandings data in the National Marine Mammal Strandings database, along with annual reports determining causes of mortality for large whales in US waters (Henry et al., 2023), allows patterns in strandings and threats to be assessed quantitatively through time.

Examining available data for large whales on the US East Coast with an eye to the ecology, population trends, and current threats to key species provides important context for recent patterns in strandings, and affords an opportunity to contribute to the management of protected species. As biologists studying whales on the East Coast who are not involved with the strandings response, we conducted an independent and informed assessment of strandings and MSI data. We examined threats to large whales, assessed spatial and temporal trends in strandings and MSI with publicly available data, and considered possible ecological and anthropogenic drivers of these patterns. Finally, we summarized the status of knowledge—and gaps in knowledge—on whale biology and causes of recent stranding events.

## VESSEL STRIKES AND ENTANGLEMENT

Vessel strikes and entanglements in fishing gear are major threats facing large whales globally and are significant causes of mortality in large whale populations (Berman‐Kowalewski et al., 2010; Knowlton et al., 2012; van Der Hoop et al., 2013; van der Hoop et al., 2015, 2017). Vessel strikes can cause mortality or serious injury in large whales, including hemorrhaging and bone fracture (Douglas et al., 2008; Knowlton et al., 2012). Mortality is more likely when vessels are traveling at high speed (Conn & Silber, 2013; Laist et al., 2001; Rockwood et al., 2020; Vanderlaan & Taggart, 2007) and in regions with high vessel traffic (van Der Hoop et al., 2012). Large ships (>80 m) are thought to be responsible for most large whale mortalities or serious injuries, and in many cases, whales are not observed prior to impact (Laist et al., 2001; Wiley et al., 2016). Entanglement in fishing gear at depth can cause the drowning when the whale is entangled, such that it cannot reach the surface to breathe, or can cause long‐term injury or mortality (Moore & van der Hoop, 2012). Whales with chronic entanglements (i.e., when fishing lines or gear remain attached over long periods) can become emaciated due to decreased feeding efficiency and increased drag or can experience infection or severe tissue damage due to lacerations from fishing lines (Moore & Van der Hoop, 2012; Moore et al., 2013a; Sharp et al., 2019; Winn et al., 2008).

## RECENT INCREASES IN LARGE WHALE STRANDINGS

**FIGURE 1:**
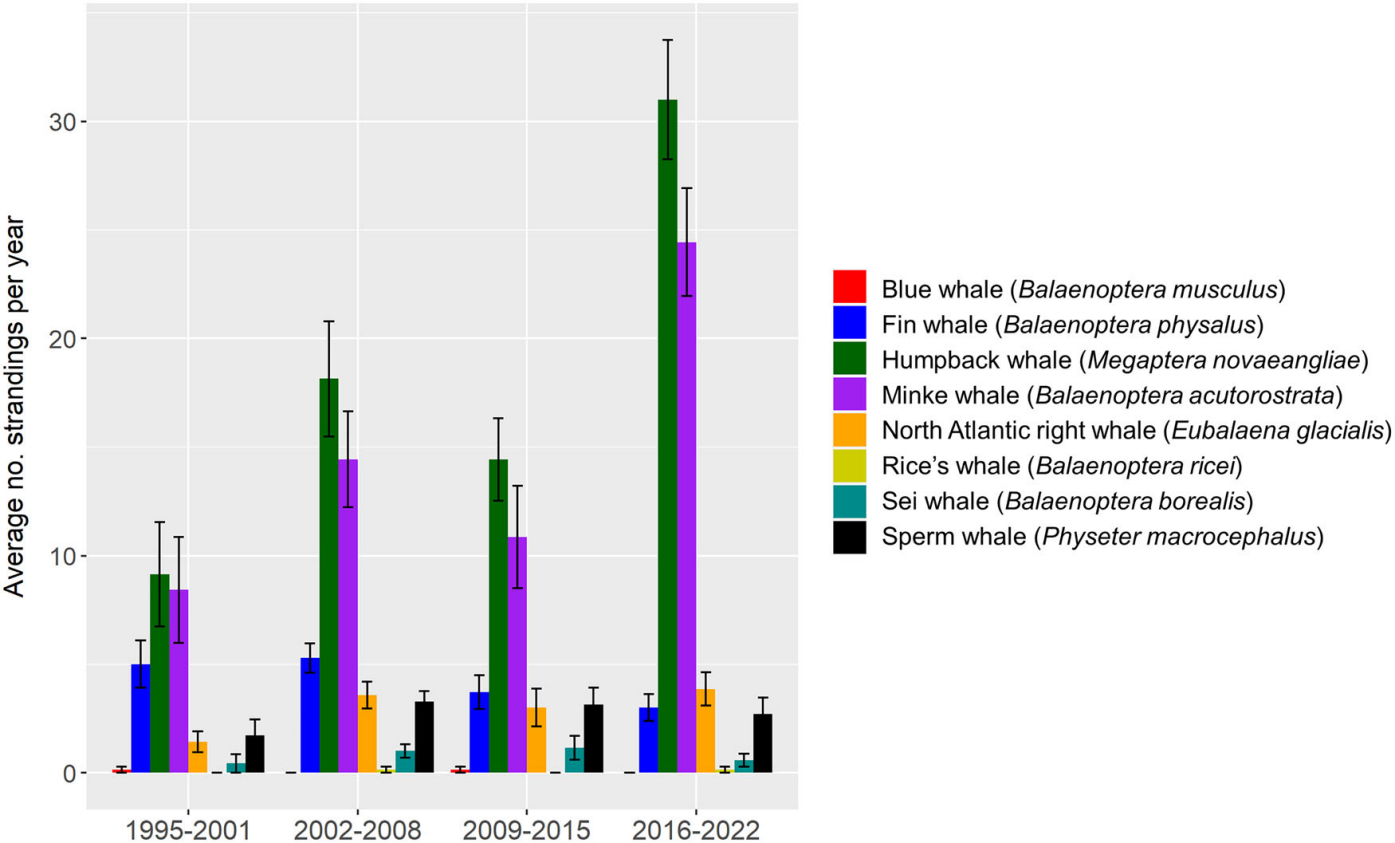
Average number of large whale strandings by species per year (+/‐ SE) along the eastern seaboard of the United States from 1995 to 2022.

Large whale strandings along the US East Coast are dominated by humpback whales (*Megaptera novaeangliae*) and minke whales (*Balaenoptera acutorostrata*) (Figure 1 & Appendix S1). Numbers of large whale strandings on the Eastern Seaboard of the United States have been elevated since 2016, resulting in the National Marine Fisheries Service (NMFS) declaring unusual mortality events (UME) for humpback, minke, and right whales (*Eubalaena glacialis*) (NMFS, 2023). Under the US MMPA, a UME is defined as “a stranding that is unexpected; involves a significant die‐off of any marine mammal population; and demands immediate response.”

Large whales differ considerably in their prey species and habitat use, making it difficult to assess patterns of strandings in space and time when examining large whale species in aggregate. Because humpbacks strand more often than other large whales in this region, we, therefore used humpback whales as a case study to assess spatiotemporal changes in strandings and to consider possible drivers of these strandings.

## HUMPBACK WHALES ON THE US EAST COAST

Humpback whales are ubiquitous and wide‐ranging baleen whales that occur from the tropics to high‐latitude waters globally. As accessible nearshore inhabitants that are easy to identify, humpback whales are among the best‐studied species of large whale (Clapham, 2000). Humpback whales were historically targeted by commercial whaling, leading to drastic reductions in population size (Best, 1993; Clapham et al., 1999). Globally, hundreds of thousands of humpback whales were harvested in the 20th century, and many populations of humpback whales may have been reduced by more than 90% (Clapham et al., 1999). Humpback whales observed along the US East Coast are part of the West Indies Distinct Population Segment (DPS) (NMFS, 2016), which breed in the West Indies and feed in foraging areas in the Northeast United States, eastern Canada, Greenland, and Iceland during summer and fall months (Clapham et al., 1993; Martin et al., 1984; Palsbøll et al., 1997; Stevick et al., 2003). This DPS has recovered following the moratorium on commercial whaling to the extent that it was delisted from the US Endangered Species Act in 2016 (Bettridge et al., 2015).

## SPATIOTEMPORAL PATTERNS IN HUMPBACK WHALE STRANDINGS

**FIGURE 2:**
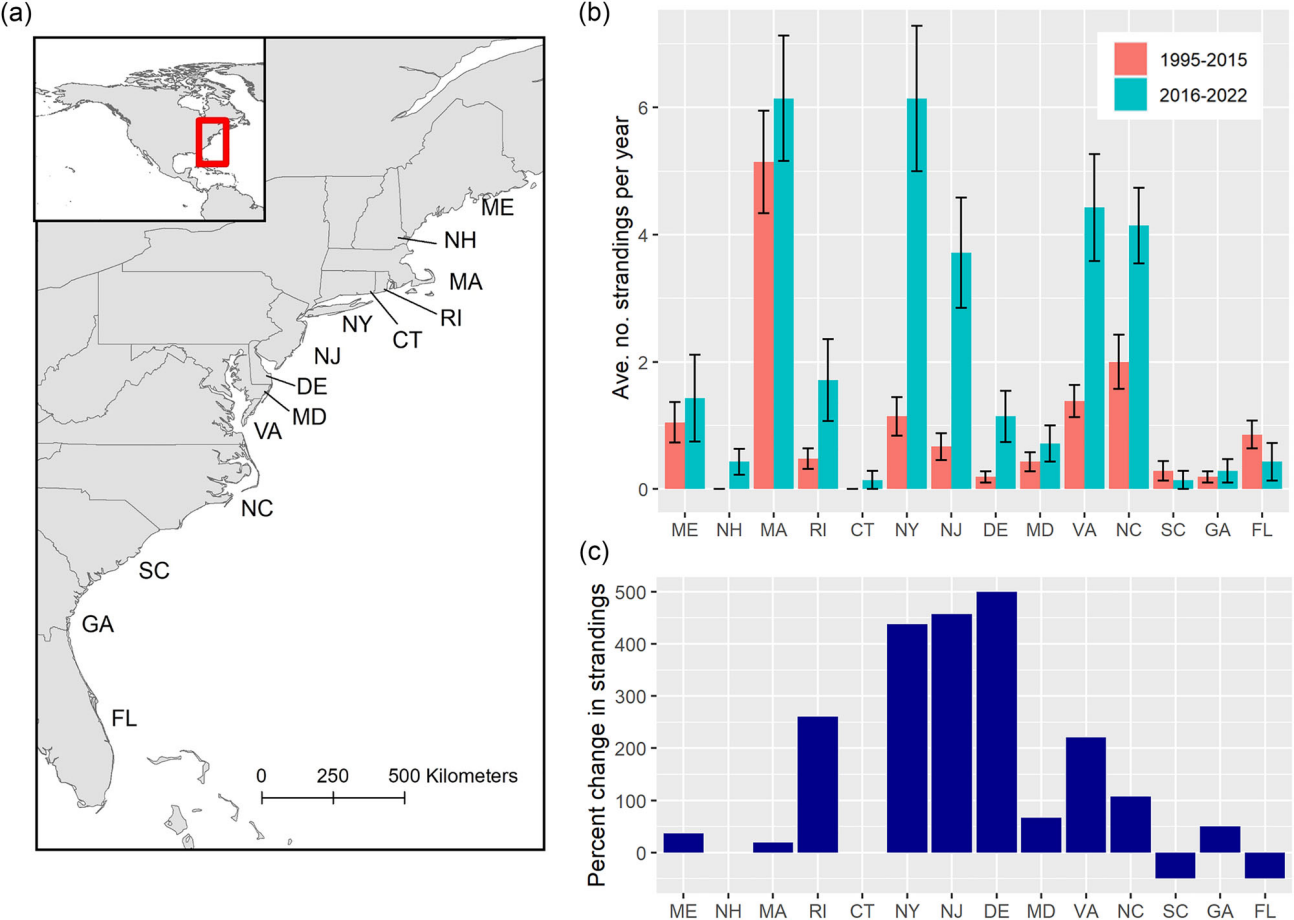
(a) Location of states along the East Coast of the United States, (b) average number of humpback whale strandings (+/‐ SE) per year by state prior to 1995−2015 and during the unusual mortality event (UME) (2016−2022), and (c) percent change in humpback whale strandings by state during UME in relative to previous years (1995−2015) (ME, Maine; NH, New Hampshire; MA, Massachusetts; RI, Rhode Island; CT, Connecticut; NY, New York; NJ, New Jersey; DE, Delaware; MD, Maryland; VA, Virginia; NC, North Carolina; SC, South Carolina; GA, Georgia; FL, Florida).

**FIGURE 3:**
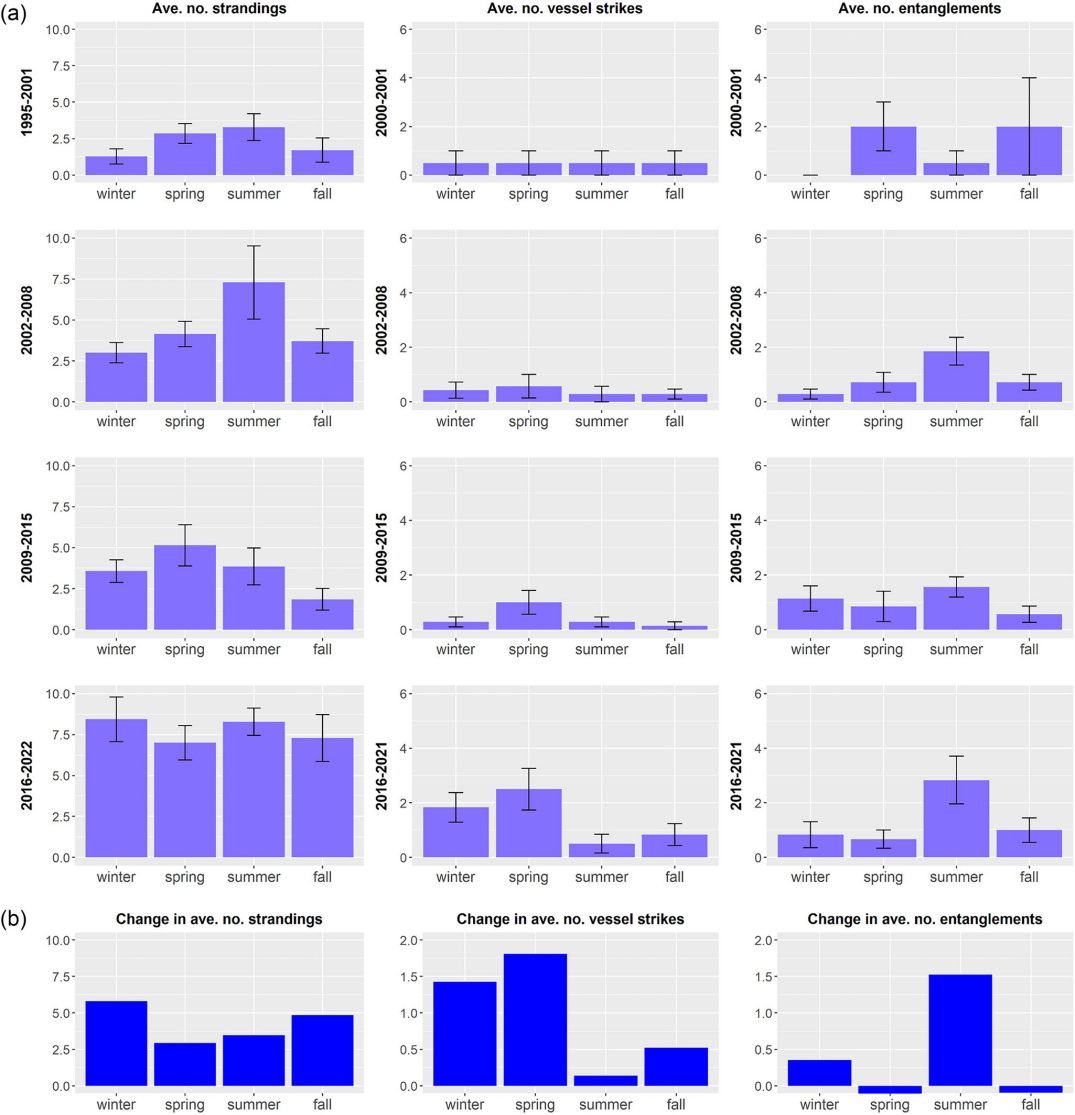
(a) Average annual number of humpback whale mortalities, strandings, and serious injuries (MSI) determined to be due to vessel strike or entanglement in fishing gear by season (+/‐SE) along the eastern seaboard of the United States in the same 7‐year periods as in Figure 1 (the first period represents annual averages from 1995 to 2001 for strandings, but only 2000−2001 for vessel strikes and entanglements due to data availability) and (b) changes in these metrics during the humpback whale unusual mortality event (2016−2022) relative to prior periods (1995−2015 for strandings data and 2000−2015 for vessel strikes and entanglements).

The ongoing UME of humpback whales began in 2016, and a comparison of humpback whale strandings per year during this period with those in prior years highlights several notable changes. First, while the number of humpback whale strandings was consistently high in Massachusetts, which has a long coastline and includes Cape Cod where many strandings occur, mid‐Atlantic states from Virginia through New York showed the greatest increases in strandings during the UME (Figure 2b,c). When adjusting for the length of coastline in each state (Appendix S1), the number of strandings during the UME was highest in mid‐Atlantic states (Appendix S2). Second, prior to 2008, strandings occurred primarily in the spring, summer, and fall, when humpback whales forage in the waters of the Northeast United States in large numbers. However, strandings began to be observed in winter more frequently from 2009 to 2015. During the UME, high numbers of strandings were observed throughout the year rather than in summer and fall, when humpback whales typically forage in high densities in the Northeast United States (Figure 3). Winter had the greatest increase in the average number of strandings per year during the UME in comparison with prior periods (Figure 3b).

## ROLE OF VESSEL STRIKES AND ENTANGLEMENTS IN RECENT LARGE WHALE STRANDINGS

Only approximately 20% of humpback whale mortalities are observed (Hayes et al., 2020). However, examining known causes of recorded MSI can provide insight into factors driving strandings. The NMFS has established criteria for determining whether MSI was due to entanglement or vessel strike based on indications, such as the presence of constricting fishing line, subdermal hemorrhaging, hematoma or edema, skeletal fracturing, or carcasses found on the bow of a ship (Henry et al., 2023). These determinations can be made conclusively for a subset of large whale strandings. Reports on MSI in baleen whales are available back to 2000 in publicly available annual reports (Appendix S1).

**FIGURE 4:**
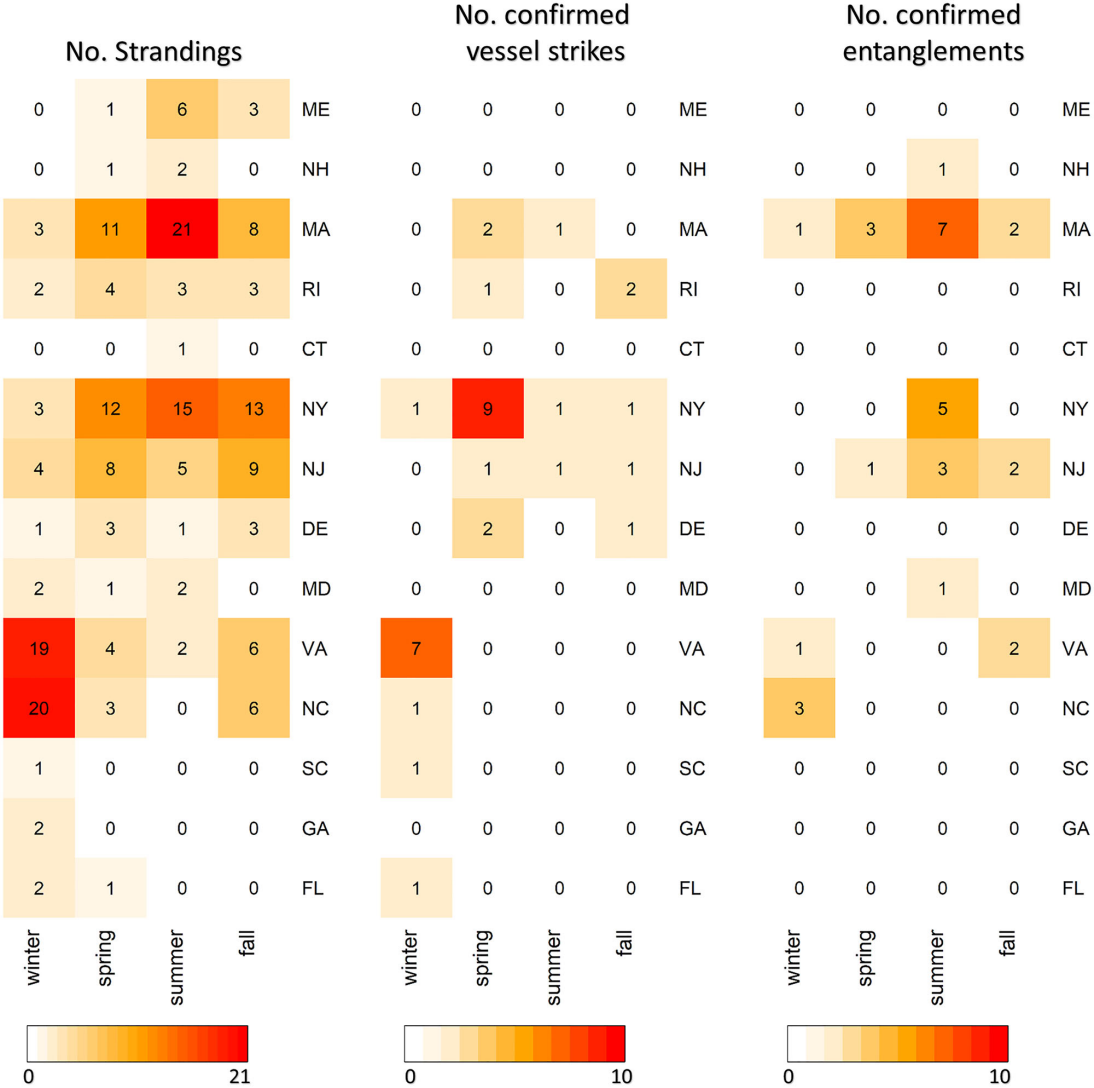
Number of humpback whale strandings, and mortalities, and serious injuries determined to be due to vessel strike or entanglement in fishing gear, respectively, by season and state during the ongoing humpback whale unusual mortality event. Due to data availability, the number of strandings reflects data from 2016 to 2022, and the number of mortalities and serious injuries determined to be due to vessel strike or entanglement in fishing gear reflects data from 2016 to 2021. The cause of death can only be determined for a subset of mortalities.

With these data, we conducted *t* tests to compare annual MSI due to vessel strikes and those due to entanglements (detailed methods in Appendices S1 & S3). Prior to the UME, humpback whale MSI due to entanglements were observed significantly more often than those determined to be due to vessel strikes (hereafter MSI due to entanglements or vessel strikes for brevity) (*p* = 5.22×10^−4^). During the UME, there was no significant difference in humpback whale MSI due to entanglements in comparison with those due to vessel strikes (*p* = 0.84). The MSI due to vessel strikes showed significant changes (3‐fold increase during the UME), and MSI due to entanglements increased, but not significantly (annual MSI for 2000−2015 vs. 2016−2022 for data throughout the East Coast: *p* = 1.46×10^−2^ for vessel strikes, *p* = 9.70×10^−2^ for entanglements) (Appendix S3 & S5). During the UME, increased MSI due to vessel strikes primarily occurred in the winter and spring, whereas those due to entanglement primarily occurred in the summer (Figure 3). Patterns of strandings mirror these findings. During the UME, humpback whale strandings during summer generally occurred in states where high numbers of mortalities or serious injuries due to entanglement were observed in those seasons (Figure 4). During winter and spring, strandings were highest in states that showed relatively higher MSI due to vessel strike during those seasons.

**FIGURE 5:**
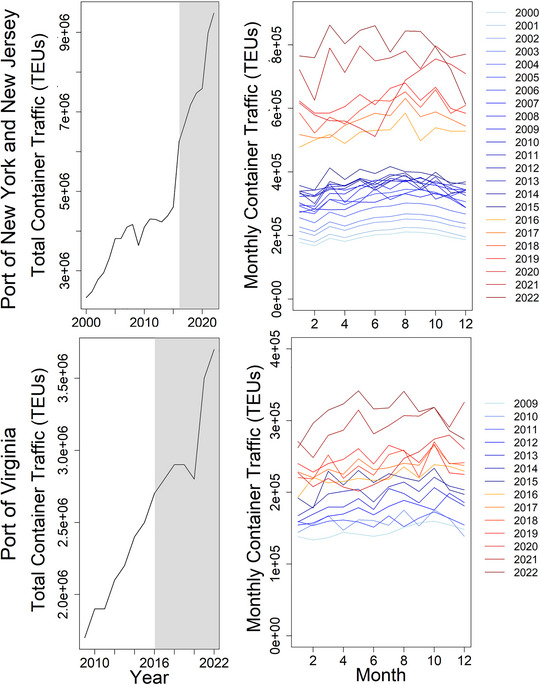
Annual and monthly container traffic at the Port of New York and New Jersey from 2000 to 2022 and at the Port of Virginia from 2009 to 2022 in 20‐foot equivalent units (TEUS). Data from the Port Authority of New York and New Jersey and from the Virginia Port Authority (as available) (gray, years during humpback whale unusual mortality event [UME]; blue lines, years prior to the onset of the UME; yellow and red lines years during the UME).

New York and Virginia were notable hotspots of vessel strikes (Figure 4), and showed the greatest increases in MSI due to vessel strikes (Appendices S5 & S6a). Container vessel traffic was much higher in these 2 states in comparison with other states in the Northeast United States (Appendix S7) and increased substantially since the onset of the UME in 2016, particularly since 2020 (Figure 5). In 2022, the Port of New York and New Jersey became the busiest port in the United States (LaRocco, 2022). Increases in vessel traffic in New York and Virginia were part of postpandemic changes in patterns of shipping in which the flow of trade has moved from the West Coast to the East Coast (LaRocco, 2022). Further, New York is a major port along shipping routes that occur adjacent to the coasts of Massachusetts and Rhode Island and routes that transit the US continental shelf south of New York and New Jersey (Notteboom et al., 2022; Stone, 2022). Thus, increased vessel traffic in the Port of New York and New Jersey would also lead to increased vessel traffic along the coasts of states such as Rhode Island, Massachusetts, and Delaware. This could explain why increases in MSI due to vessel strike have been observed in these states, used by humpback whales for foraging or migratory habitat, even though ports in these states have not shown notable increases in vessel traffic (Appendices S5, S6a, & S7). Together, spatiotemporal patterns in MSI suggest that the increase in strandings of humpback whales in New York, New Jersey, and Virginia and proximate states during the UME is likely attributable, at least in part, to increased vessel traffic leading to increased vessel strikes.

## ADDITIONAL FACTORS INFLUENCING ELEVATED STRANDINGS AND VESSEL STRIKES IN MID‐ATLANTIC STATES

Although increases in strandings occurred throughout mid‐Atlantic states, increases in MSI due to vessel strikes have been particularly marked in New York and Virginia (Figures 2 & 4, Appendices S5 & S6a). In addition to increases in vessel traffic, changes to humpback whale habitat use may play a role in the observed increases in strandings and MSI. The estimated abundance of humpback whales in the Gulf of Maine has increased, although relatively slowly, over the past 20 years (Hayes et al., 2020; Robbins & Pace, 2018). Although the Gulf of Maine was previously considered the southernmost summer feeding ground used regularly by humpback whales in the Northwest Atlantic (Barco et al., 2002), humpback whales have been observed foraging regularly in waters of New York and New Jersey since approximately 2011 (Brown et al., 2018; King et al., 2021; Smith et al., 2022; Stepanuk et al., 2021). Additionally, humpback whales have been observed frequently in the mid‐Atlantic region of the United States in recent decades (New York to Virginia) during winter months (Barco et al., 2002; Wiley et al., 1995).

The regular use of summer foraging habitat in New York waters likely represents an expansion of humpback whales into other foraging grounds as the abundance of humpback whales in the Gulf of Maine has increased. Increases in inshore Atlantic menhaden (*Brevootia tyrannus*) may also play a role in the increase in humpback whale sightings in the mid‐Atlantic states (Brown et al., 2018). The increase in strandings in mid‐Atlantic states corresponds with an increase in the spawning stock biomass of Atlantic menhaden forage fishes, during that same period. Menhaden are a small, energy‐rich forage fish targeted by humpback whales and many recent observations of humpbacks feeding in the mid‐Atlantic region identify menhaden as prey (e.g., Brown et al., 2018; Lomac‐MacNair et al., 2022). Previously overfished, menhaden abundance in the mid‐Atlantic region began to increase in the mid‐2010s, with spawning aggregations occurring in the mid‐Atlantic region (Simpson et al., 2017; Atlantic States Marine Fisheries Commission, 2022).

Menhaden tend to form dense surface schools in shallow coastal waters in mid‐Atlantic states (Brown et al., 2018; Goetsch et al., 2023). Humpback whales foraging in Virginia and New York waters often use surface foraging behavior (Smith et al., 2022; Stepanuk et al., 2021; Swingle et al., 1993), which may make whales more vulnerable to vessel strike (Parks et al., 2012; Wiley et al., 2011a). In addition, foraging in shallow nearshore waters of New York and Virginia (Brown et al., 2019; King et al., 2021; Stepanuk et al., 2021; Swingle et al., 1993) may make humpback whales more likely to be struck by vessels approaching the coast (Laist et al., 2001). Further, humpback whales foraging in the coastal waters of New York and Virginia are predominantly juveniles (Barco et al., 2002; Brown et al., 2022; Stepanuk et al., 2021; Swingle et al., 1993). Juvenile whales may be more susceptible to anthropogenic threats due to a lack of experience (Lien, 1994). In fact, juvenile humpback whales make up the vast majority of observed humpback whale strandings on the East Coast of the United States (Stepanuk et al., 2021; Wiley et al., 1995).

These factors suggest that the increase in vessel strikes in New York and Virginia is likely due to the combined effects of changes in humpback whale habitat use, surface feeding behavior, occurrence in shallow waters, prevalence of juvenile whales, and increases in vessel traffic in these regions (Figure 5).

## VESSEL STRIKE FREQUENCY IN WINTER AND SPRING

Humpback whales have been observed year‐round throughout regions of the Northeast United States, though at lower densities during winter months (Clapham et al., 1993; Davis et al., 2020; Zoidis et al., 2021; Zoodsma et al., 2016), suggesting that some whales are not migrating to the West Indies breeding grounds. Increases in vessel strikes in winter and spring during the 2009−2015 and 2016−2022 periods, particularly in New York and Virginia (Figures 3 & 4), occurred despite similar levels of vessel traffic year‐round in these states (Figure 5). The waters of coastal Virginia are thought to serve as a supplemental feeding ground during winter months (Barco et al., 2002; Swingle et al., 1993; Wiley et al., 1995). The increase in strandings and vessel strikes in New York in spring (Figure 4) could reflect migrating whales, with whales in this foraging area at higher risk of vessel strike due to surface foraging behavior as described in the previous section. Although humpback whales are frequently resighted in New York waters throughout the summer months (Brown et al., 2022), humpbacks observed in New York in spring may be part of a migratory pulse to more northerly foraging grounds.

## ROLE OF OFFSHORE WIND DEVELOPMENT IN LARGE WHALE STRANDINGS

Although there are many offshore wind projects currently undergoing permitting and assessment in the Northeast United States, very little offshore wind energy development occurred during the study period (through 2022). There were only 2 operational offshore wind farms along the US East Coast during this time frame: one off Block Island in Rhode Island, consisting of 5 offshore wind turbines, and one off the coast of Virginia, consisting of 2 wind turbines. These turbines represent a total of 42 MW of energy, whereas the potential energy capacity of wind energy areas undergoing approvals, permitting, site controls, and planning, as well as the potential for regions yet to be leased, is estimated to be 52,687 MW (NREL, 2023) (Appendix S8).

Cetaceans rely heavily on sound for communication, navigation, foraging, and predator avoidance (Tyack, 1997), and anthropogenic noise can alter behavior, increase stress, and inhibit communication in large whales (Hatch et al., 2008, 2012; Madsen et al., 2006; Van Parijs et al., 2021). Offshore wind development has led to concern regarding the impacts of associated anthropogenic sound on these species (Bailey et al., 2014; Dolman & Simmonds, 2010; Madsen et al., 2006). Potential impacts of offshore wind development occur in 3 phases of development (site assessment and characterization, construction, and operation) and may be important in the assessment of cumulative impacts of multiple ecosystem stressors associated with future offshore wind development. Wind energy development activities in any of these phases that may potentially affect marine mammals must be authorized by the US Fish and Wildlife Service or the National Oceanic and Atmospheric Administration and must meet requirements for monitoring and mitigating harm to these species under the MMPA.

### Site assessment and characterization

Site assessment and characterization surveys of offshore wind activities are conducted prior to construction. We focused in more detail on this phase of development because this was the phase that predominantly occurred during the study period.

Sounds produced by boomers, sparkers, bubble guns, and subbottom profilers used during site assessment and characterization surveys overlap with the humpback whale hearing range (Appendix S9). A list of the sound characteristics of these sources is in Appendix S10 (Ruppel et al., 2022). Multibeam echosounders and sidescan sonars are used in site characterization surveys on the US East Coast, but their frequency ranges are almost exclusively above the humpback whale hearing range (Crocker et al., 2018; Ruppel et al., 2022). Air guns are not being used for offshore wind surveys on the US East Coast (Baker & Howson, 2021). Air‐gun arrays are of concern for baleen whales because of their high source level and because peaks in the power spectrum of this sound source overlap with the estimated hearing range of baleen whales (Nowacek et al., 2007) and baleen whales have shown behavioral responses to air guns (e.g., Castellote et al., 2012; Di Iorio & Clark, 2010; Dunlop et al., 2015, 2017).

The majority of research assessing the acoustic impacts of offshore wind development on cetaceans has focused on construction given the low frequencies and relatively high sound energy levels of activities during this phase of development (e.g., pile driving [Bailey et al., 2010; Madsen et al., 2006; Thomsen et al., 2006]). Sound sources used in site assessment and characterization surveys typically have lower sound energy levels and higher frequencies (Appendices S9 & S10) and have thus received less attention to date. Absorption of sound is frequency dependent, with high‐frequency sounds being absorbed more rapidly than lower‐frequency sounds as distance from the source increases (Urick, 1983). As a result, the potential impacts of higher‐frequency sound sources on cetaceans (e.g., injury or behavioral responses) are constrained to short distances (Ellison et al. 2012). A small number of recent assessments provide insight into the potential impacts of site assessment and characterization surveys on baleen whales and determined that these surveys were expected to have minimal behavioral effects given the small ranges over which behavioral impacts would be expected (Baker & Howson, 2021; Ruppel et al., 2022) (Appendix S9; Thomsen et al., 2006).

Authorizations from NMFS are required for site assessment and characterization surveys, and these publicly reported authorizations provide a coarse metric of when and where these surveys occur. We examined the year and state in which humpback whale strandings and authorizations for site assessment and characterization surveys occurred during the UME (2016−2022) (Appendix S1). The resulting spatiotemporal patterns did not suggest a link between strandings site assessment and characterization surveys (Appendix S11). Survey authorizations increased over the course of the UME and primarily occurred between New Jersey and Massachusetts, but elevated numbers of strandings did not follow this pattern and predated the survey authorizations. For example, 2016 included only 1 survey authorization related to offshore wind, in Massachusetts, but elevated numbers of strandings were observed from North Carolina to Rhode Island, whereas Massachusetts showed a lower number of strandings in 2016 relative to the years prior to the UME. Although some increases in strandings occurred in years and states with multiple survey authorizations (e.g., in 2018‐2022 in New York, 2022 in New Jersey), many did not. For example, elevated numbers of strandings were observed in Virginia and North Carolina in 2016 and 2017 when there were no survey authorizations either in these states or in neighboring states. The largest increase in strandings in New Jersey was observed in 2019, when no surveys were authorized in this state.

### Construction of offshore wind farms

The construction of offshore wind farms may pose a threat to whales and other marine mammal species, primarily due to noise associated with pile driving (Bailey et al., 2010; Dähne et al., 2013; Dolman & Simmonds, 2010, 2010; Madsen et al., 2006; Thompson et al., 2010, 2020). Although the UME is ongoing, only 7 wind turbines were constructed during the 2016−2022 period of the UME analyzed here. Construction of the 5 wind turbines at Block Island in Rhode Island took place from 2015 to 2016 (Carey et al., 2020), whereas the 2 turbines off the coast of Virginia were constructed in 2020 (VOWDA, 2021). The highest numbers of humpback strandings in Rhode Island and neighboring states took place in years after construction occurred (e.g., 2017, 2022), whereas the highest numbers of humpback whale strandings in Virginia were observed prior to 2020 when construction in this area took place (Appendix S11). Thus, the timeline and spatial extent of offshore wind construction does not line up for the construction of offshore wind farms to be a driver of the humpback whale strandings.

### Operation of offshore wind farms

During operation, offshore wind farms produce low noise levels that are not likely to impair hearing in cetaceans (Madsen et al., 2006; Tougaard et al., 2009). Although vessel strikes associated with construction and maintenance vessels from offshore wind development may pose a risk (Bailey et al., 2014), only a small number (7) of wind turbines were operational during the study period, representing minimal vessel traffic.

### Cumulative impacts of ecosystem stressors associated with offshore wind development

Although our findings suggest that wind energy activity is unlikely to have been a driver of the recent increase in humpback whale strandings, noise from site construction, operation, and decommissioning, and shipping traffic to and from sites will each contribute additional sound in an already noisy and compromised marine environment.  Increased vessel traffic could also increase the direct risk of whale mortality through collision, though increases in vessel traffic associated with wind energy development are likely to be small relative to the very high vessel densities occurring in mid‐Atlantic states. Beyond direct impacts on large whales, an understanding of the influence of permanent, widespread, and large‐scale wind farm development, such as the impact on ocean currents, reduction in water column stratification, and deflection of the pycnocline (National Academies of Sciences, Engineering & Medicine, 2023), each of which are important to local productivity, should be a priority. The cumulative impacts of these stressors associated with future offshore wind development should be assessed.

## GAPS IN KNOWLEDGE OF WHALES AND VESSEL STRIKES

Our findings suggest that vessel strikes are a key driver of the recent increase in large whale strandings along the US East Coast and highlight important gaps in knowledge regarding this issue. First, a more thorough understanding of the movements and habitat use of humpback and other large whales, both spatially and seasonally, is needed to better understand and predict threats to these species. Second, assessments of broad spatiotemporal changes to the abundance and distribution of prey species that might influence large whale distribution are needed to understand both the habitat use and health of large whales. Third, more work is needed to determine how habitat use and foraging behavior (e.g., surface feeding and feeding in shallow coastal habitats) might affect the risk of vessel strike.

In addition to these knowledge gaps, there are many open questions regarding factors directly influencing vessel strikes. For example, what size and class of vessels is involved in vessel strikes, particularly in New York and Virginia? Are there aspects of whale foraging behavior that put large whales at particular risk of vessel strike? Are vessels in New York and Virginia adhering to regulations that limit the vessel speed in seasonal management areas (SMAs), established to reduce vessel collisions with North Atlantic Right Whales? Are SMAs, designed to protect right whales, also providing protection for other large whales such as humpbacks, or are species‐specific SMAs needed? Are there feasible changes to vessel regulations that could further decrease interactions? Improving knowledge of these factors is necessary to better understand the drivers of large whale mortalities and to develop effective strategies to mitigate the impacts of anthropogenic activities in coastal and offshore waters.

## CONCLUSIONS

Our findings point to vessel strikes, exacerbated by increased vessel traffic in new foraging areas used by naïve, juvenile humpback whales, as a major driver of recent increases in strandings and MSI in humpback whales along the Eastern Seaboard of the United States. We found no evidence that offshore wind development in the Northeastern United States played a role in observed patterns of strandings and MSI. Thus, our findings suggest that mitigation measures focused on reducing vessel strikes, which result directly from human activities and threaten large whales globally, should be a priority in order to decrease large whale mortality. Our findings highlight the extent to which changes in global shipping patterns and changes in large whale habitat use can shift hotspots of risk and the need for management actions that can respond to the dynamic nature of such threats. Mitigation measures have been established to reduce the risk of vessel strikes on North Atlantic right whales based on seasonal habitat use, and our assessment points to the need to consider additional management actions that would offer protection to other large whale species. Further, managers need to assess the cumulative impacts of wind farms, including potential impacts on oceanography and productivity, and consider specific policy actions, such as speed restrictions in vessels serving wind farms. The results of our analyses support the urgent need for best practice mitigation and monitoring methods needed to support adaptive management in a region that is already heavily affected by anthropogenic threats. This work highlights the need for, and importance of, rigorous, ongoing, long‐term data collection for large whale strandings to aid management decisions. Public interest in the welfare of marine mammals is high, emphasizing the need to assess and communicate fact‐based information on the drivers of large whale mortality.

## Supporting information

Supporting Information
