# Supplementary material for: Evaluating drivers of recent large whale strandings on the East Coast of the United States
Source: Conserv Biol. 2024 May 29;38(6):e14302. doi: 10.1111/cobi.14302 (PMC11588986; doi:10.1111/cobi.14302)
Supplement: Supplementary file 1 — Supporting Information [file COBI-38-e14302-s001.docx]

**Appendix SI: Data sources and methods**

*Strandings, mortalities and serious injuries of humpback whales*

Data on large whale strandings from 1995-2022 along the east coast of the US were obtained from the National Marine Mammal Stranding Database. Details on public access to the data are outlined at <https://www.fisheries.noaa.gov/national/marine-life-distress/national-stranding-database-public-access>

We provide an overview of the species composition of large whales stranding on the eastern seaboard of the US through time (Figure 1 in the main text), but the remainder of the paper focuses on humpback whales specifically. We included floating strandings of humpback whales in our analysis (i.e., those that did not wash up on shore, but which were assessed at sea). While floating strandings were typically described within the database as being within the US Exclusive Economic Zone (EEZ) rather than within a particular state, we assigned these strandings to states using the description of the location of the stranding (e.g., “22 miles east of Cape May Inlet”) and/ or the latitude and longitude for the stranding.

Data on the mortalities and serious injuries of humpback whales were obtained from annual reports of human-caused mortality and serious injury (MSI) to baleen whale stocks along the Gulf of Mexico, United States East Coast, and Atlantic Canadian Providences produced by the Northeast Fisheries Science Center (<https://repository.library.noaa.gov/>). These reports provide detailed data on MSI of baleen whales dating back to 2000, with the most recent report providing data through 2021. Since mortality and serious injury determinations require detailed assessment and review, the availability of these data trails that of strandings data by a year. The cause of many large whale MSI cannot be determined conclusively as they cannot be linked with tell-tale physical evidence. A variety of factors, such as decomposition, chronic entanglement, and post-mortem marks or lesions can complicate efforts to distinguish cause of death (Moore et al. 2013). Determinations of MSI are based on the best available information (Henry et al. 2023)

We examined spatial and temporal patterns in MSI along the US east coast from 2000-2021, using entries categorized as a mortality or serious injury due to vessel strike or entanglement. Under the Marine Mammal Protection Act, human-caused MSI is compared to the Potential Biological Removal (PBR) to determine if a stock is considered strategic, and each mortality and serious injury counts as a value of 1 against PBR. For data from 2007 and on, poorly documented injury events are prorated (assigned a value between 0 and 1) based on available information and observed ratios of mortality to survival (Cole and Glass 2013, Henry et al. 2023). We included only serious injuries assigned a value of 1 in our analyses (i.e., we did not include prorated injuries) to allow for a consistent analysis from 2000-2021 since prorated injuries were not included in reports prior to 2007.

When assessing patterns of strandings and MSI, we assessed the number of strandings by kilometer of coastline in each state (Wiley et al. 1995) since the amount of coastline could influence the likelihood that whales strand on shore and are observed. We created a shapefile of ocean-facing coastline in each state (e.g., excluding coastline in the Long Island Sound and Chesapeake Bay) from the US states basemap in ArcGIS Desktop 10.8.1 and quantified the distance of ocean-facing coastline in each state using the Spatial Analyst toolbox in ArcMap (ESRI 2021).

*Other data sources*

Data on monthly cargo volumes at the Port of New York and New Jersey were obtained from the Port Authority of New York and New Jersey website (<https://www.panynj.gov/port/en/our-port/facts-and-figures.html> ). Monthly cargo volumes at the Port of Virginia were obtained from the Virginia Port Authority website (<https://operations.portofvirginia.com/port-statistics> ). These detailed monthly data were available from 2000-2022 for New York/ New Jersey, and from 2009-2022 for Virginia.

Annual data on container vessels from 2003-2021 from ports throughout the US East Coast were obtained from the “US waterborne container traffic by port/ waterway by year” data set, accessed from the US Army Corps of Engineers Digital Library: <https://usace.contentdm.oclc.org/digital/collection/p16021coll2/id/12774/rec/1>

Annual landings from the American Lobster fishery were obtained from NOAA Fisheries Commercial Fisheries Statistics: <https://www.fisheries.noaa.gov/national/sustainable-fisheries/commercial-fisheries-landings> .

Following the US Marine Mammal Protection Act, Incidental Harassment Authorizations (IHAs) are required for marine site assessment and site characterization surveys for renewable energy development that have the potential to result in harassment to marine mammals. Data on IHAs for renewable energy activities under the Marine Mammal Protection Act were obtained from NOAA fisheries: <https://www.fisheries.noaa.gov/national/marine-mammal-protection/incidental-take-authorizations-other-energy-activities-renewable>. We examined all IHAs related to wind energy development during the 2016-2022 UME. For example, in 2016, an IHA pertaining to geophysical and geotechnical surveys in offshore Massachusetts was considered in our analysis since it focused on surveys to characterize the Massachusetts Lease Area, while IHAs pertaining to the Port and Algonquin pipeline operation, maintenance and repair in Massachusetts Bay and to the decommissioning of Neptune Deepwater Port in Massachusetts were not. We summed the number of IHAs by state and year to assess spatiotemporal patterns in IHAs, including any states listed in the Federal Register for the survey authorization. For example, the IHA for the Atlantic Shores Offshore Wind Bight, LLC site characterization surveys included waters off the coast of New York and New Jersey in 2022 and 2023. In our analysis, this IHA was recorded as including a survey in both of these states in both 2022 and 2023. In some instances, the title of the authorization did not include all of the states indicated in the Federal Register; for example, the survey authorization for Avangrid Renewables LLC in the area of OCS-A508 only mentions coastal North Carolina, but the Federal Register describes surveys taking place in both Virgina and North Carolina, and both states were therefore included in the analysis.

One survey authorization in Virginia, for the Coastal Virginia Offshore Wind Commercial (CVOW Commercial) Project in 2020 and 2021, was not listed on the NOAA fisheries website, but details were obtained from the Federal Register for inclusion in this analysis. When renewals of survey authorizations occurred, we only added additional years beyond the initial authorization into the summary of survey authorizations by year and state. For example, if the initial authorization included covered 2018 and 2019, and the renewal listed 2018, 2019 and 2020, only 2020 was added to the analysis (on top of the initial authorization). We note that in several instances, wind energy companies changed names and the initial authorization therefore included a different name than the renewal. For example, an initial survey authorization for surveys in New York waters was issued to Statoil in 2018, while the renewal of the same survey lists the companies new name, Equinor, in 2019. In other instances, renewals for survey authorizations noted that surveys had not yet been initiated due to delays; in these instances only the years represented in the renewals were included in the analysis. For example, in 2020 NOAA Fisheries published a proposed survey authorization for Mayflower Wind Energy LLC in waters off of Massachusetts, but the final IHA was not issued as the company determined that they needed to modify their survey plans. A modified survey authorization was issued in 2021, and 2020 was not included in the analysis.

**
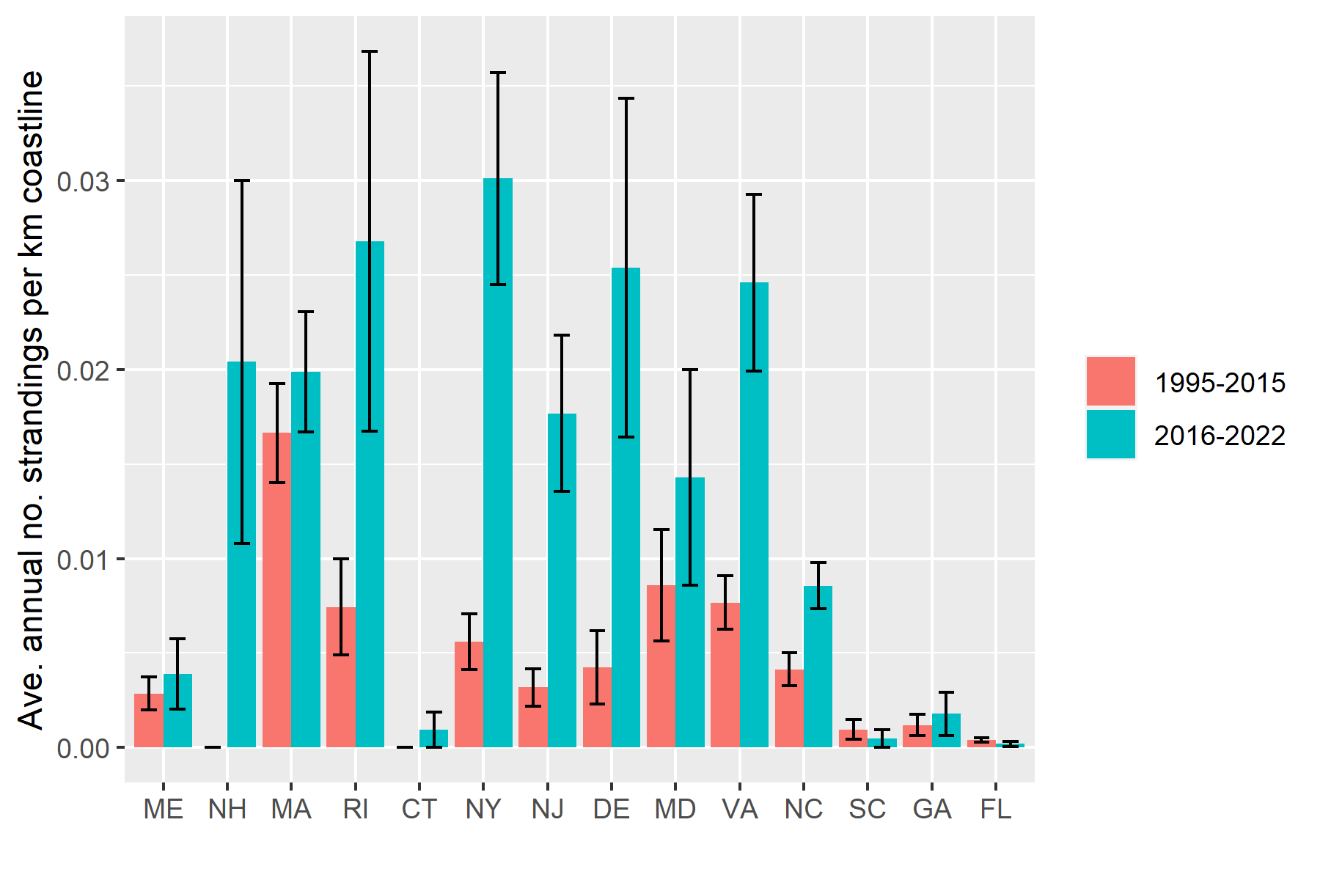
**

**Appendix S2**: Average number of humpback whale strandings (+/- SE) per year and kilometer of coastline in each state on the US eastern Seaboard prior to (1995-2015) and during the Unusual Mortality Event for humpback whales (2016-2022). State abbreviations are as follows: ME= Maine, NH= New Hampshire, MA= Massachusetts, RI= Rhode Island, CT= Connecticut, NY= New York, NJ= New Jersey, DE= Deleware, MD= Maryland, VA= Virginia, NC= North Carolina, SC= South Carolina, GA= Georgia, FL= Florida.

**Appendix S3: Patterns of humpback whale entanglements during the ongoing Unusual Mortality Event**

Increases in entanglements during the UME occurred during the summer when humpback whales are typically foraging in high densities in the Northeast US (Figure 3 in main text). Lobster or crab pots/ traps and gillnets are the fishing gear types most frequently involved in humpback whale entanglements in the Northeast US (Johnson et al. 2005). While the state of Maine has by far the highest lobster fishing landings in the Northeast US (Appendix S4), states from Massachusetts to Virginia showed the highest MSI due to entanglements during the UME (Figure 4 in main text, Appendix S6b). New York and New Jersey showed increases in MSI due to entanglements during the UME (Appendix S6b). No MSI due to entanglements was observed in Maine during the UME. Lobster landings increased markedly in Maine from 2009-2015, but other states did not show substantial increases in landings during the UME. Gillnet fishing effort occurs in coastal regions throughout the Northeast US (Orphanides 2009, Orphanides and Palka 2013), but many fish species are caught using gillnets (Orphanides 2009, Bisack and Das 2015) and changes to fisheries landings associated with gillnets is therefore harder to assess. Changes in the habitat use of humpback whales, or in the spatial distribution of fishing effort, or both, could conceivably cause more overlap between whales and fishing operations and could therefore play a role in the observed increase in MSI due to entanglements in New York and New Jersey. However, an important caveat to strandings caused by entanglement is that large whales can swim considerable distances once entangled and the site of stranding might not be the site of actual entanglement. Thus, animals entangled in areas such as Maine or Canada might ultimately succumb and strand in other locations, such as the mid-Atlantic.


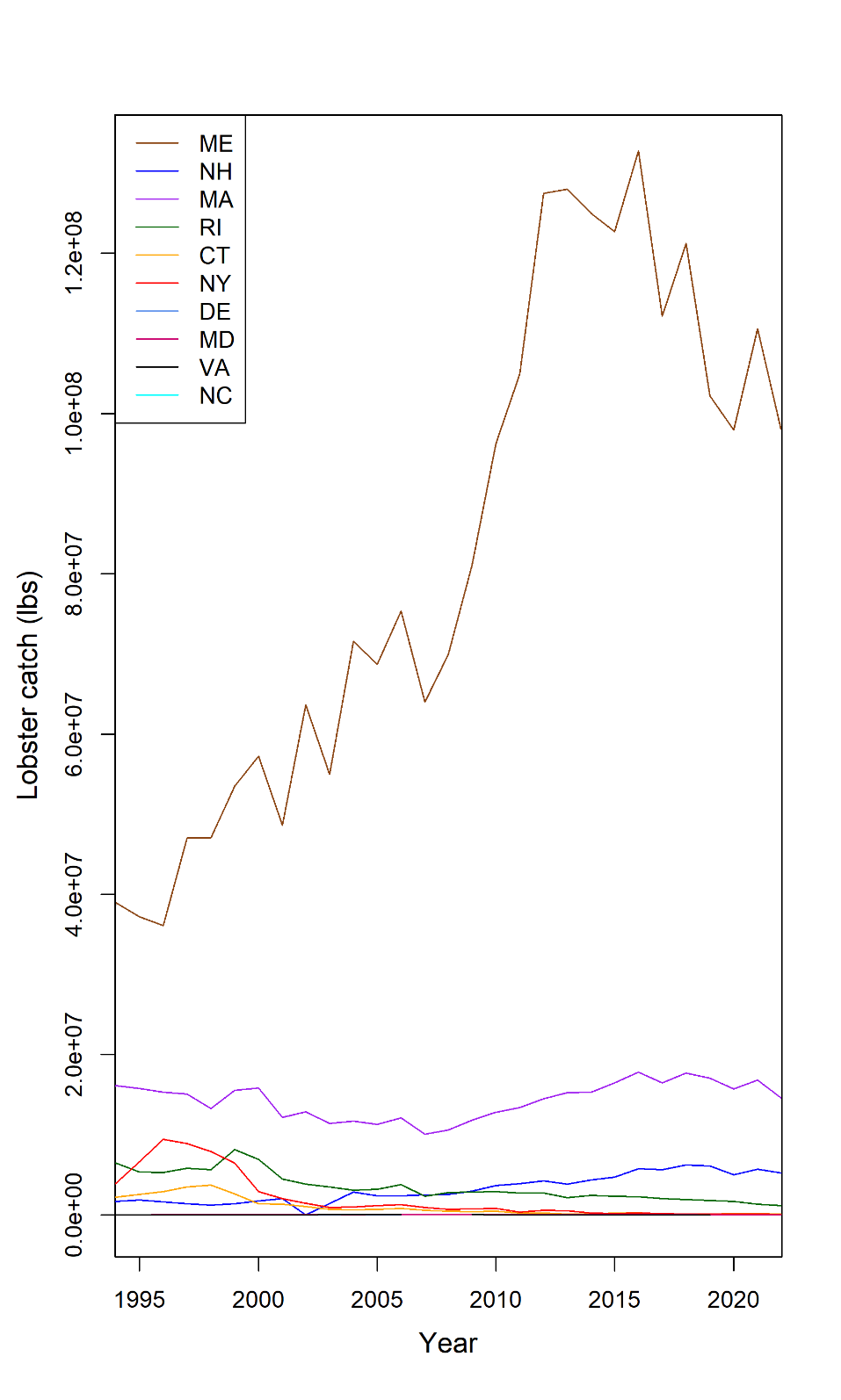


**Appendix S4:** Annual landings by state from the commercial fishery for American lobster from 1995-2022. Data from NOAA Fisheries Commercial Fisheries Statistics.


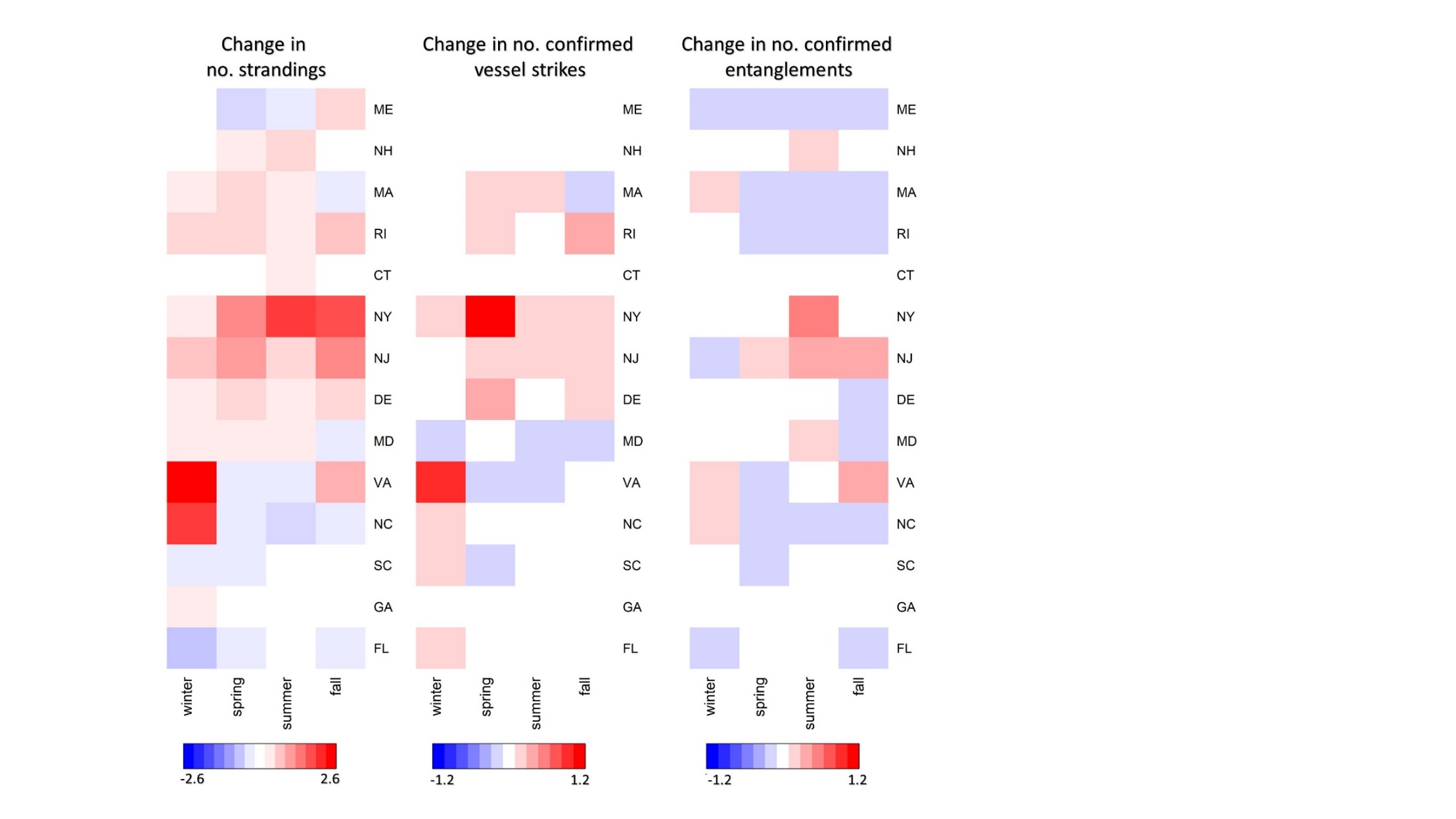


**Appendix S5:** Differences in the following parameters between the ongoing humpback whale Unusual Mortality Event (UME; 2016-2022 for strandings, 2016-2021 for mortalities and serious injuries) and prior time periods (2000-2015) by season and state: number of humpback whale strandings, mortalities and serious injuries determined to be due to vessel strike, and those determined to be due to entanglement in fishing gear. State abbreviations are as in Appendix S2.


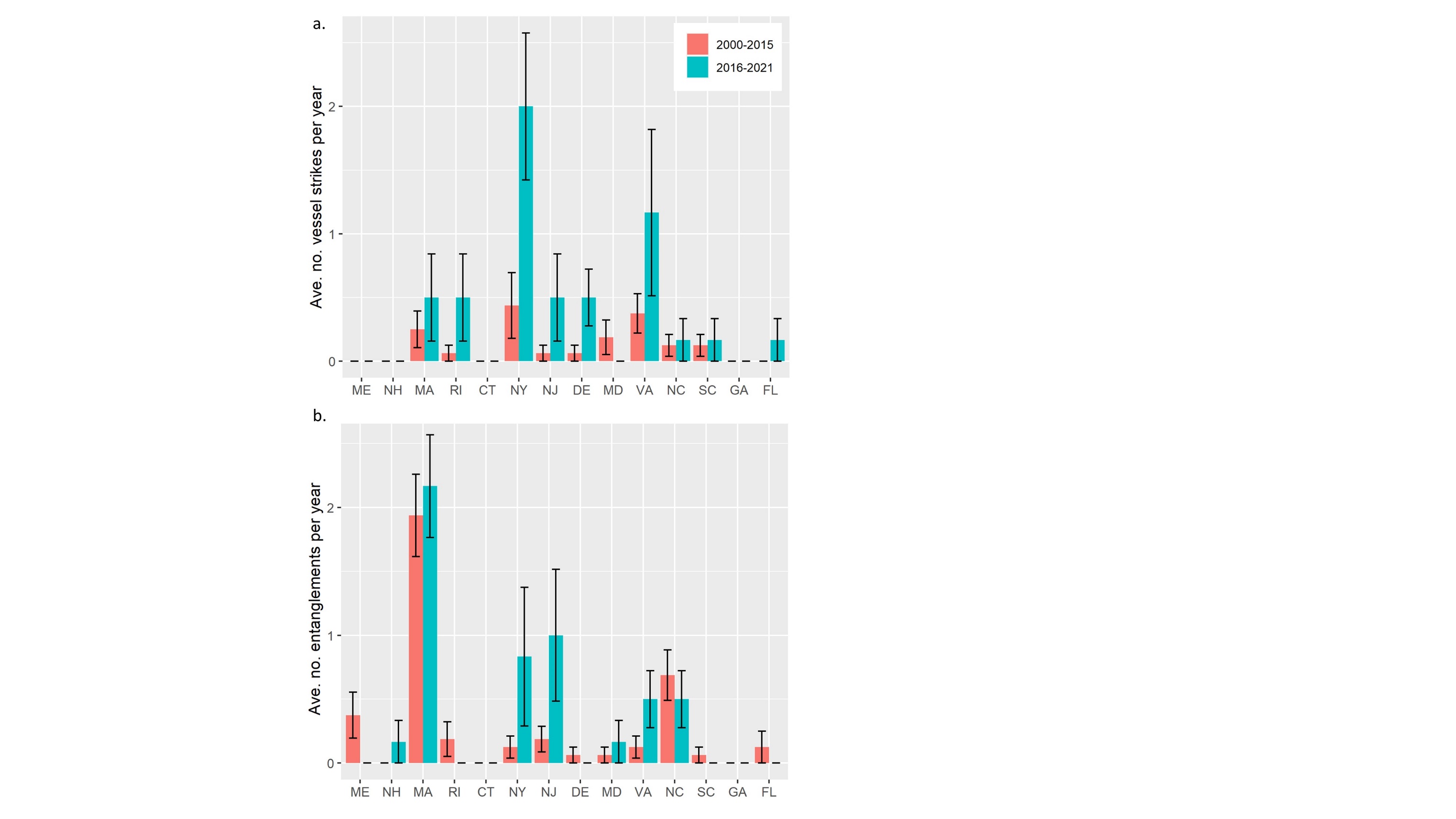


**Appendix S6**: Average number of humpback whale mortalities and serious injuries per year (+/- SE) (a) determined to be due to vessel strikes and (b) determined to be due to vessel strikes by state prior to (2000-2015) and during the Unusual Mortality Event for humpback whales (data from 2016-2021). State abbreviations are as in Appendix S2.


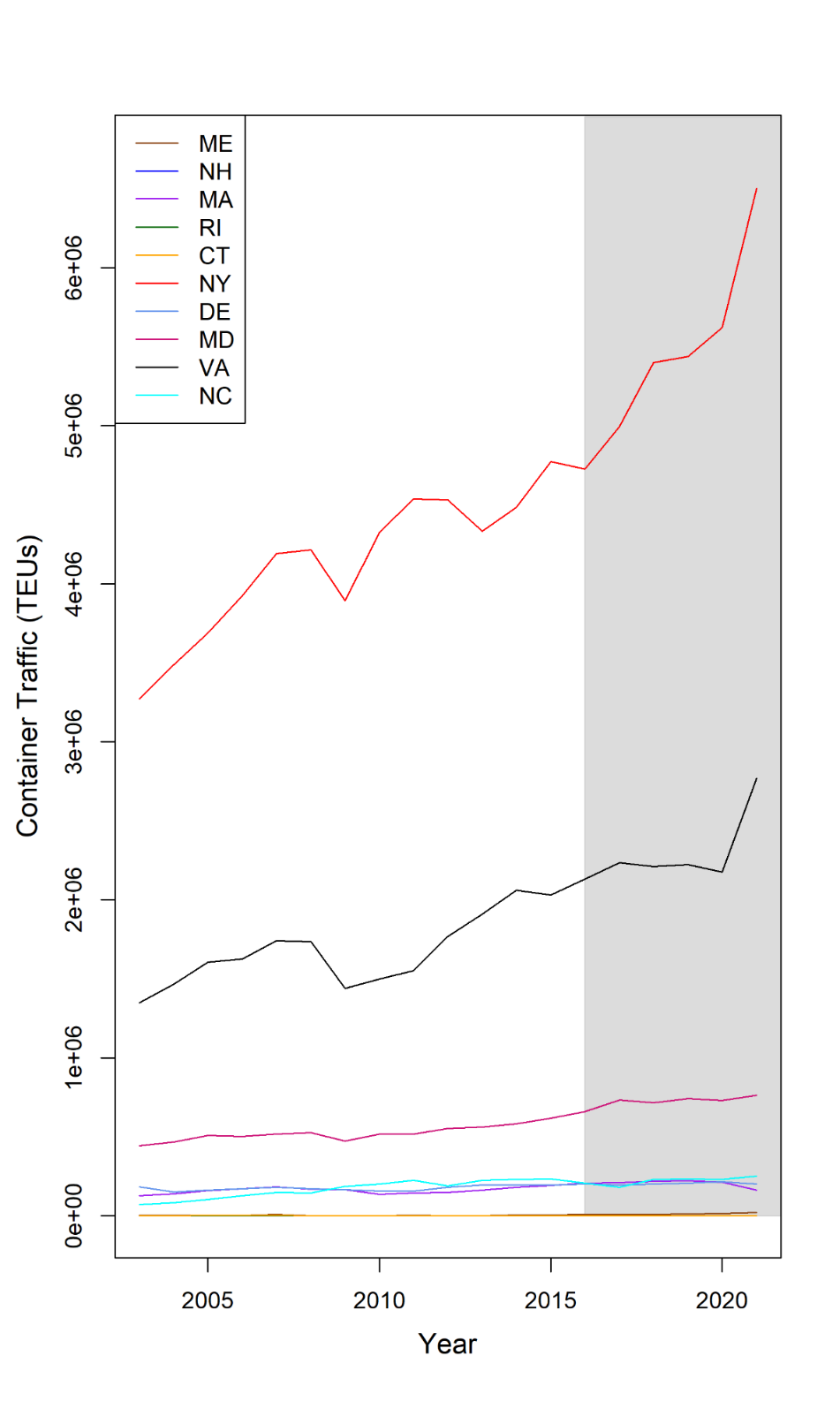


**Appendix S7**: Container vessel traffic in Twenty-Foot Equivalent Units (TEUs) at shipping ports in states within in the Northeast US from 2003-2021. The period of the ongoing humpback whale Unusual Mortality Event is shown in gray. State abbreviations are as in Appendix S2. Data from the US Army Corps of Engineers Digital Library. We note that the TEUs shown here for New York and Virginia differ from those used taken from the Port of New York and New Jersey (2000-2022), and from the Port of Virginia from (2009-2022) in Figure 5 of the main text due to reporting differences between these sources. We use data in this figure as a means of comparing across states using the same metrics and reporting criteria.


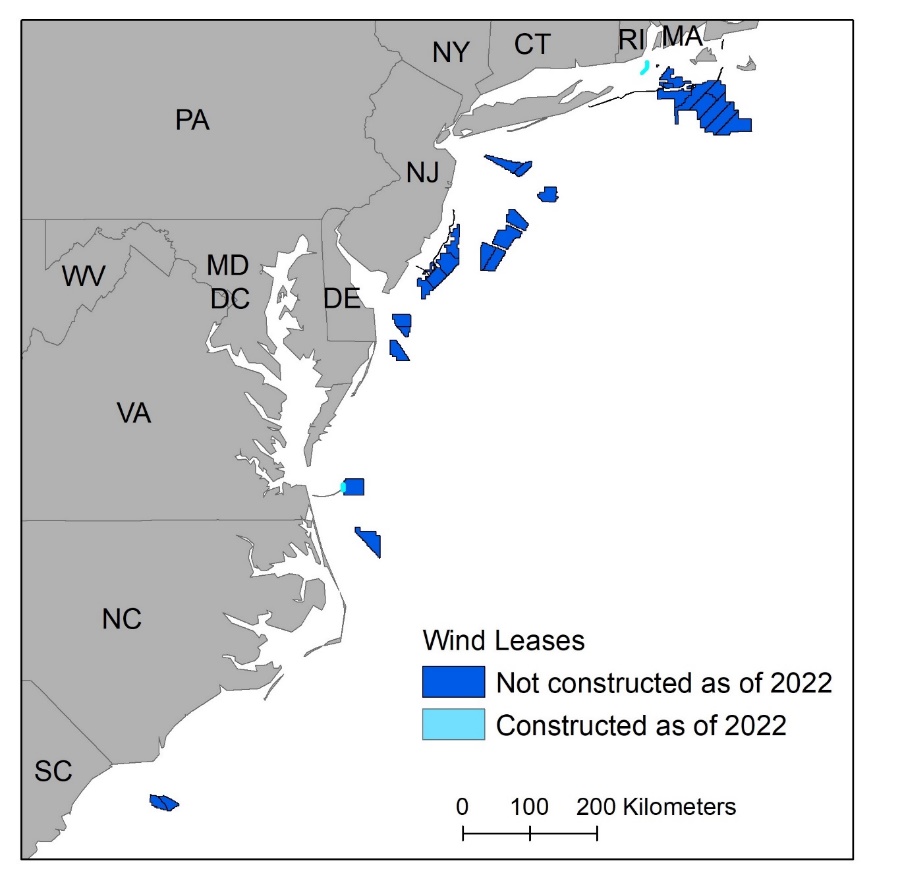


**Appendix S8:** Wind energy leases along the eastern seaboard of the US that were constructed as of 2022 and those that were in stages of planning or development but were not constructed as of 2022.

**Appendix S9: Current knowledge of acoustic impacts of site characterization and assessment surveys**

*Humpback whale hearing*

Humpback whales produce sounds that typically range in frequency from approximately 20 Hz to 4 KHz, with high frequency harmonics reaching 24 kHz (Winn and Winn 1978, Au et al. 2006, Kowarski et al. 2019). Anatomical studies suggest their best hearing sensitivity is between approximately 200 Hz and 9 kHz (Houser et al. 2001, Tubelli et al. 2018).

*Prior studies assessing site assessment and characterization surveys*

Baker and Howson (2021) evaluated the risk of Permanent Threshold Shift (PTS), or the permanent reduction in sensitivity to sound, and disturbance for baleen whales based on sound source characteristics of surveys used during site assessment and characterization. PTS was estimated to occur over distances of less than 13 m from the source, while the largest worst-case behavioral distances were 502 m. Thomsen et al. (2023) modeled the acoustic impacts of sparkers used during offshore wind surveys on minke whales, and also found low threshold distances (up to 200 m for PTS and up to 1100 m for Temporary Threshold Shift (TTS), or the temporary reduction in sensitivity to sound, and behavioral response). Given the low spatial densities of baleen whales, these sources were expected to have minimal effects. Ruppel et al. (2022) assessed detailed characteristics of geophysical surveys and classified survey types into tiers according to their likelihood or resulting in behavioral takes under the MMPA (harassment). Survey types that are used during site assessment and characterization in the US were either determined to be unlikely to result in the incidental take of marine mammals (sub-bottom profilers and low-powered sparkers) or required additional data or modeling to determine conclusively whether they fall into this category (boomers and some sparker configurations). Importantly, none of the survey types used in site assessment or characterization that overlap with the estimated humpback whale hearing range were categorized as potentially causing behavioral takes of marine mammals in this study. We note that which sounds are classified as “High-resolution Geophysical Sources” varies between studies and we therefore avoid using this term herein.

**Appendix S10:** Characteristics of sound sources used during marine geophysical surveys (from Ruppel et al. 2021). Sound sources used in site characterization and assessment on the US east coast that are within the range of best hearing sensitivity for humpback whales are shown in bold. We note airguns are not used during site assessment and characterization on the US east coast.

| **Marine Acoustic Source** | **Transmission frequency** | **Source Level (dB re 1 uPa @ 1m)** | **Max pulse duration (ms)** | **Min. Ping Repetition rate (s)** |
| --- | --- | --- | --- | --- |
| Single airgun (seismic) | 15–60Hz | 216–235 | Few ms | >5s |
| Airgun arrays (seismic) | 15–60Hz | 228–259 | Few ms | >5s |
| **Boomer (seismic)** | **300–3000Hz** | **185–207** | **0.6** | **0.167** |
| **Sparker (seismic)** | **300–1400Hz** | **185–226** | **3** | **0.25** |
| **Bubble gun (seismic)** | **20–2000Hz** | **194–220** | **1.6** | **0.125** |
| **Hull-mounted sub-bottom profilers** | **3.5,12kHz** | **199–232** | **64** | **1** |
| **Shallow-towed sub-bottom profilers** | **0.5–24kHz** | **146–180** | **9** | **0.125** |
| **Parametric sub-bottom profilers** | **1–115kHz** | **206–247** | **2.5** | **0.025** |
| Multibeam echosounder | 12–600kHz | 175–245 | 100 | 5 |
| Sidescan sonar | 65–500kHz | 196–224 | 0.4–1.6 | 0.013 |

**
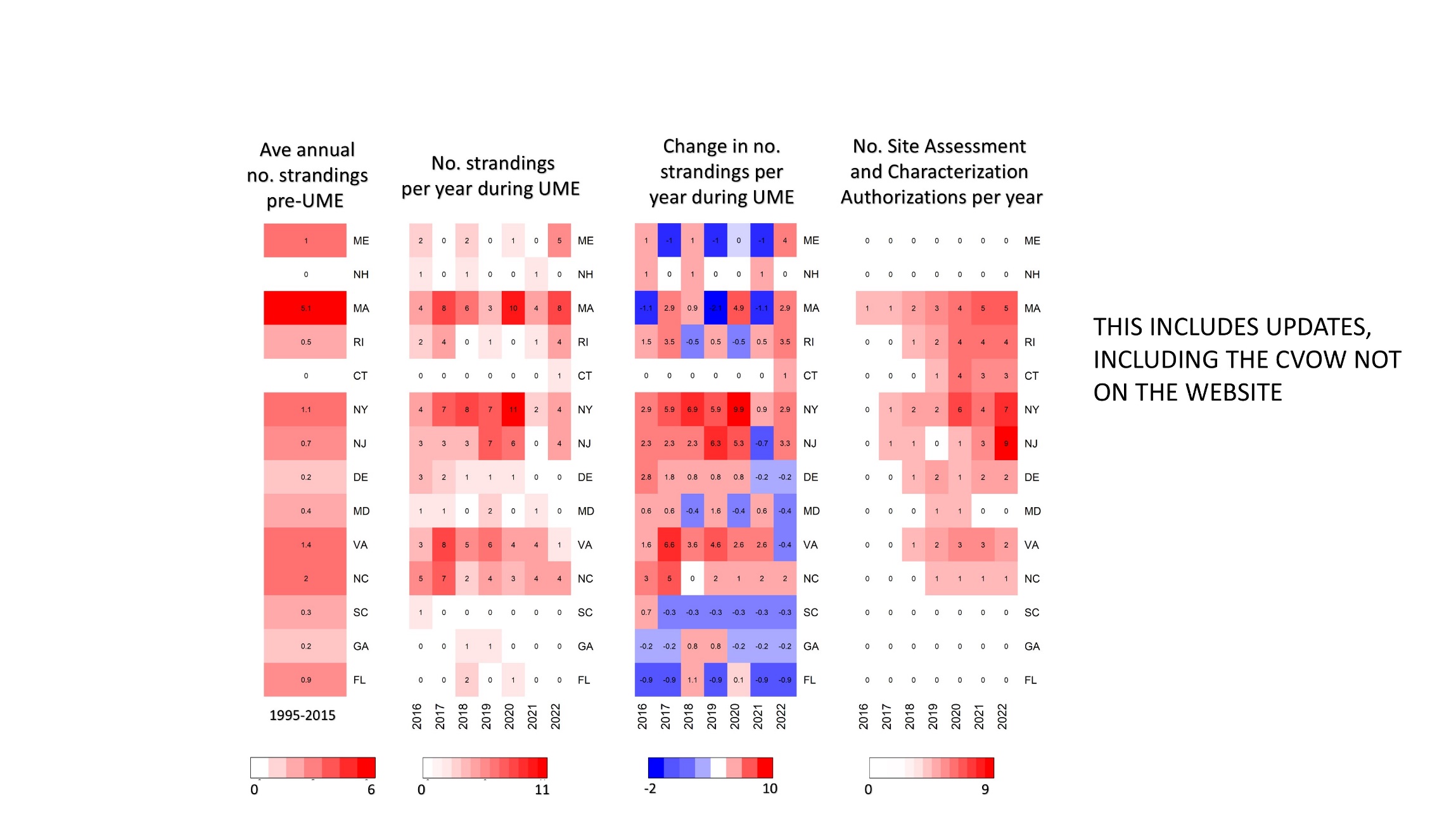
**

**Appendix S11:** Spatiotemporal patterns in humpback whale strandings and site characterization surveys on the US east coast. Data are as follows: the average number of humpback whale strandings occurring prior to the ongoing humpback whale UME (data from 1995-2015); the number of strandings observed by state and year during the UME (2016-2022); changes in the number of strandings observed in each state during each year of the UME in comparison to the pre-UME annual average for that state; and number of site assessment and characterization authorizations per year and state. Authorization data were obtained from NOAA fisheries: <https://www.fisheries.noaa.gov/national/marine-mammal-protection/incidental-take-authorizations-other-energy-activities-renewable>

**References**

Au WW, Pack AA, Lammers MO, Herman LM, Deakos MH, Andrews K. 2006. Acoustic properties of humpback whale songs. The Journal of the Acoustical Society of America 120: 1103–1110.

Baker K, Howson U. 2021. Data Collection and Site Survey Activities for Renewable Energy on the Atlantic Outer Continental Shelf Biological Assessment. US Department of the Interior, Bureau of Ocean Energy Management, Office of Renewable Energy Programs.

Bisack KD, Das C. 2015. Understanding non-compliance with protected species regulations in the Northeast USA gillnet fishery. Frontiers in Marine Science 2: 91.

Cole TV, Glass AH. 2013. Serious injury determinations for Baleen whale stocks along the Gulf of Mexico, United States east coast, and Atlantic Canadian provinces, 2007-2011.

ESRI. 2021. ArcGIS Desktop: Reslease 10.8.1.

Henry A, Garron M, Morin D, Smith A, Reid A, Ledwell W, Cole T. 2023. Serious injury and mortality determinations for baleen whale stocks along the Gulf of Mexico, United States East Coast, and Atlantic Canadian Provinces, 2017-2021. Northeast Fisheries Science Center Reference Document 23–09.

Houser DS, Helweg DA, Moore PW. 2001. A bandpass filter-bank model of auditory sensitivity in the humpback whale. Aquatic Mammals 27: 82–91.

Johnson A, Salvador G, Kenney J, Robbins J, Kraus S, Landry S, Clapham P. 2005. Fishing gear involved in entanglements of right and humpback whales. Marine Mammal Science 21: 635–645.

Kowarski K, Moors-Murphy H, Maxner E, Cerchio S. 2019. Western North Atlantic humpback whale fall and spring acoustic repertoire: Insight into onset and cessation of singing behavior. The Journal of the Acoustical Society of America 145: 2305–2316.

Moore M, van der Hoop J, Barco SG, Costidis AM, Gulland FM, Jepson PD, Moore KT, Raverty S, McLellan WA. 2013. Criteria and case definitions for serious injury and death of pinnipeds and cetaceans caused by anthropogenic trauma. Diseases of aquatic organisms 103: 229–264.

Orphanides CD. 2009. Protected species bycatch estimating approaches: estimating harbor porpoise bycatch in US northwestern Atlantic gillnet fisheries. Journal of Northwest Atlantic Fishery Science 42.

Orphanides CD, Palka DL. 2013. Analysis of harbor porpoise gillnet bycatch, compliance, and enforcement trends in the US northwestern Atlantic, January 1999 to May 2010. Endangered Species Research 20: 251–269.

Ruppel CD, Weber TC, Staaterman ER, Labak SJ, Hart PE. 2022. Categorizing active marine acoustic sources based on their potential to affect marine animals. Journal of Marine Science and Engineering 10: 1278.

Thomsen F, Ram M, Chreptowicz M, Nocoń M, Balicka I. 2023. Noise modelling and environmental risk assessment of a geophysical survey and its impact on herring and minke whales in Irish coastal waters. Marine Institute, Galway.

Tubelli AA, Zosuls A, Ketten DR, Mountain DC. 2018. A model and experimental approach to the middle ear transfer function related to hearing in the humpback whale (Megaptera novaeangliae). The Journal of the Acoustical Society of America 144: 525–535.

Wiley DN, Asmutis DP, Pitchford TD, Gannon D. 1995. Stranding and mortality of humpback whales, Megaptera novaeangliae, in the mid-Atlantic and southeast United States, 1985-1992. Fishery Bulletin 93: 196–205.

Winn H, Winn L. 1978. The song of the humpback whale Megaptera novaeangliae in the West Indies. Marine Biology 47: 97–114.
